# Exercise adherence, perceived exercise benefits and barriers, and spinal mobility in ankylosing spondylitis: a cross-sectional study

**DOI:** 10.1007/s00296-026-06118-z

**Published:** 2026-04-24

**Authors:** Ahmet Usen, Ozlem Kuculmez, Mithat Oguz Yavuz

**Affiliations:** 1 Department of Physical Medicine and Rehabilitation, Medipol University Faculty of Medicine, TEM Avrupa Otoyolu Göztepe Çıkışı No: 1, Bagcilar, 34214 Istanbul, Türkiye; 2 Department of Physical Medicine and Rehabilitation, Başkent University Alanya Hospital, Alanya, Türkiye

**Keywords:** Ankylosing spondylitis, Exercise therapy, Patient compliance, Spine, Health behavior

## Abstract

Exercise adherence is essential in the management of Ankylosing Spondylitis (AS), yet its association with perceived exercise benefits/barriers and spinal mobility is not fully understood. This study aimed to assess exercise adherence and investigate its relationships with perceived exercise benefits and barriers, spinal mobility, and clinical characteristics in patients with AS. Seventy-one patients with AS were included in this cross-sectional study. Exercise adherence was assessed using the Exercise Adherence Rating Scale (EARS). Perceived benefits and barriers were evaluated with the Exercise Benefits/Barriers Scale (EBBS). Spinal mobility was assessed using the Bath Ankylosing Spondylitis Metrology Index (BASMI). Correlation analyses and multivariable linear regression were performed to identify independent predictors of exercise adherence. The EARS total score was 33.48 ± 10.81 (median 34, range 13–59). EARS total score showed a strong positive correlation with EBBS benefits (*r* = 0.608, *p* < 0.001), while the correlation with EBBS barriers was not significant (*r* = 0.198, *p* = 0.098). In multivariable regression analysis, BASMI (β = − 1.73, *p* = 0.001), EBBS benefits (β = 0.463, *p* < 0.001), and disease duration (β = 0.423, *p* = 0.019) were independently associated with exercise adherence. In patients with AS, higher exercise adherence is independently associated with greater perceived benefits of exercise, longer disease duration, and lower spinal mobility impairment. Interventions targeting motivational factors and mobility limitations may improve adherence to prescribed exercise programs.

**Supplementary Information:**

The online version contains supplementary material available at 10.1007/s00296-026-06118-z.

## Introduction

Ankylosing spondylitis (AS) is a chronic inflammatory rheumatic disease predominantly affecting the axial skeleton, and over time it may lead to restricted spinal mobility, pain, and functional impairment [1]. The progressive and lifelong nature of the disease necessitates consideration of not only pharmacological therapies but also regular exercise interventions as a fundamental component of comprehensive management [1, 2]. Contemporary reviews emphasize that optimal axial spondyloarthritis (axSpA) management requires integration of pharmacological therapy, structured exercise, patient education, and multidisciplinary care approaches [3]. Exercise is strongly supported by evidence in the management of AS with respect to preserving spinal mobility, improving posture, reducing pain, and maintaining functional capacity [2, 4]. Accordingly, current guidelines emphasize the recommendation of individualized and sustainable exercise programs for individuals with AS [2].

Existing evidence regarding the effects of exercise interventions in AS reports favorable outcomes on pain intensity and functional capacity. However, their effects on spinal mobility are more limited and demonstrate variability in terms of clinical significance [5]. In the majority of these studies, outcomes were evaluated following short-term interventions, and the overall quality of evidence has been reported to range from low to moderate [5]. Moreover, exercise has been shown to influence inflammatory processes. A 12-week home-based walking program was reported to reduce the proportion of proinflammatory monocytes, and this reduction was associated with decreases in spinal pain and inflammatory markers [6]. Although high-intensity exercise programs are often perceived as physically and mentally demanding, they have been reported to enhance body confidence and promote positive changes in attitudes toward exercise [7]. Similarly, healthcare professionals identify high symptom burden, depression, and insufficient structured support as major barriers to maintaining recommended physical activity levels in axSpA patients [8]. Collectively, these findings indicate that exercise exerts multidimensional effects in the management of AS. Nevertheless, the role of exercise type, intensity, and long-term sustainability in determining clinical outcomes remains insufficiently clarified [5, 6]. Therefore, identifying strategies to enhance long-term exercise adherence has emerged as a critical research priority.

The sustainability of clinical benefits derived from exercise depends largely on patients’ adherence to the prescribed frequency, intensity, and duration of exercise. The World Health Organization defines adherence as the extent to which a person’s behavior corresponds with agreed recommendations in terms of frequency, intensity, and duration; in inflammatory arthritis, exercise adherence is recognized as a key determinant of optimal health outcomes, yet adherence rates remain suboptimal [9]. A recent cross-sectional survey demonstrated that although nearly 90% of patients with axSpA perceived physical activity as influencing their disease course, only 27% exercised regularly, and almost half reported at least one barrier, most commonly pain and fatigue [10]. Despite regular exercise recommendations for patients with AS/axial spondyloarthritis, a substantial proportion fail to achieve recommended levels of physical activity, and both general and disease-specific barriers negatively affect adherence [7]. Similarly, positive health beliefs regarding exercise have been associated with sustained long-term physical activity in individuals with axSpA [11]. However, psychological theories and behavior change techniques are used only to a limited extent in existing intervention studies. This has contributed to insufficient evidence regarding the maintenance of long-term exercise adherence and the persistence of important methodological gaps [9]. Although current management recommendations emphasize encouraging patients to engage in regular exercise, further investigation is warranted to elucidate the determinants of exercise adherence and their associations with clinical outcomes [2].

In this context, the primary aim of the present study was to determine the level of adherence to home-based exercise programs in patients diagnosed with AS and to examine the relationship between exercise adherence and spinal mobility. Secondarily, we aimed to evaluate the associations between exercise adherence and perceived barriers and benefits to exercise, disease activity, functional status, pain intensity, and sociodemographic characteristics.

## Materials and methods

### Study design

This study was designed as a single-center, observational, cross-sectional investigation. The findings were reported in accordance with the STROBE (Strengthening the Reporting of Observational Studies in Epidemiology) guidelines [12].

### Ethical approval

Ethical approval was obtained from the Istanbul Medipol University Non-Interventional Clinical Research Ethics Committee (17/02/2026; E10840098-202.3.02-1290 ). Written informed consent was obtained from all participants prior to enrollment. The study was conducted in accordance with the principles of the Declaration of Helsinki.

### Study setting and participants

The study was conducted at the Department of Physical Medicine and Rehabilitation, Istanbul Medipol University Faculty of Medicine. Patients presenting to the rheumatology and physical therapy outpatient clinics who met the eligibility criteria were recruited consecutively.

Patients meeting the following criteria were included:

- Age ≥ 18 years;
- Diagnosis of AS according to the modified New York criteria [13];
- Disease duration of at least 6 months;
- Previous prescription of a home-based exercise program;
- Willingness to participate and provision of written informed consent.

Exclusion criteria were: severe cardiopulmonary disease precluding exercise, history of major orthopedic surgery within the previous 6 months, active malignancy, pregnancy or lactation, cognitive impairment interfering with questionnaire completion, or uncontrolled psychiatric disorders.

### Sample size calculation

Sample size was calculated using G*Power 3.1.9 software for a multiple linear regression model in which the total EARS score was specified as the dependent variable. The sample size calculation was based on the model explanatory power (R² ≈ 0.18) derived from a pilot analysis of the first 30 enrolled patients, corresponding to a medium-to-large effect size according to Cohen’s classification (f² ≈ 0.22). Considering seven independent variables planned for inclusion in the model, with 80% statistical power (1–β = 0.80) and a type I error rate of 5% (α = 0.05), the required sample size was calculated as approximately 71 participants.

**Fig. 1:**
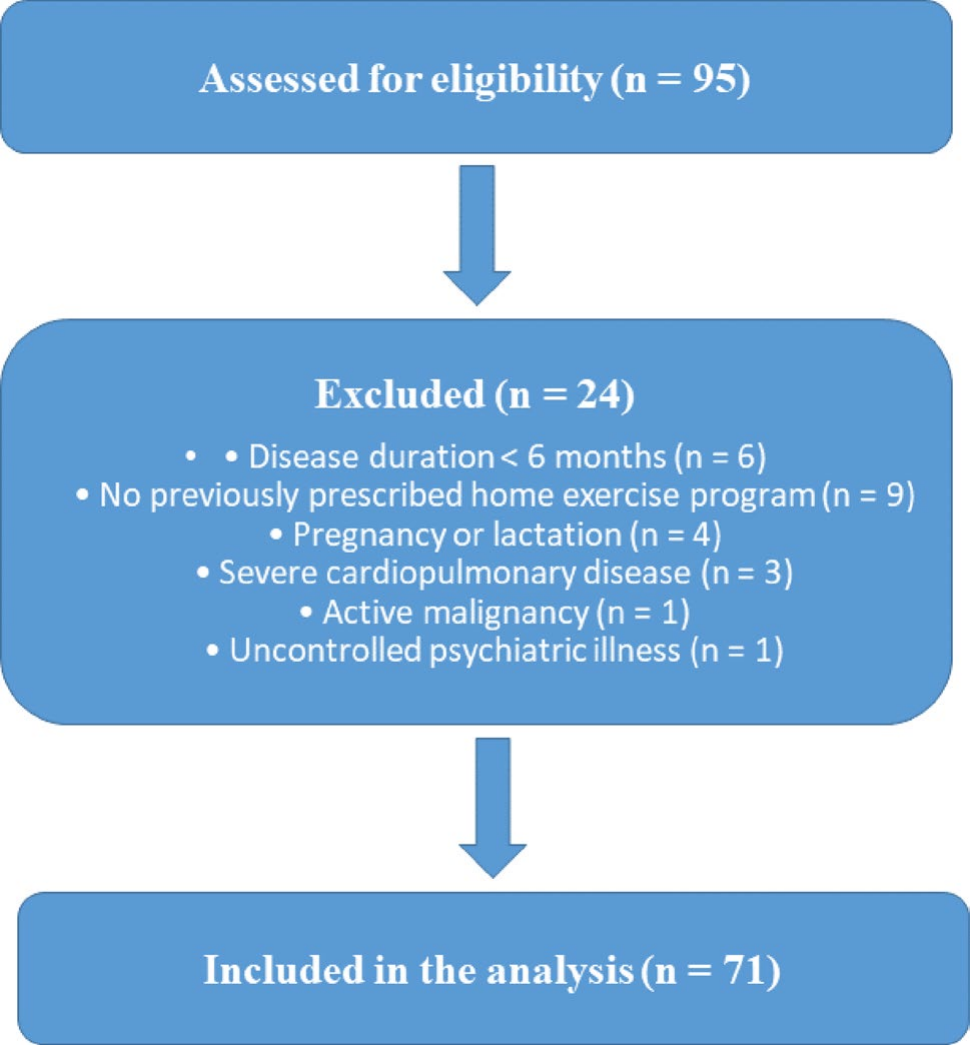
Flow diagram of participant selection and inclusion process

During the study period, 95 patients were assessed for eligibility. Six patients were excluded due to disease duration of less than 6 months, nine due to absence of a previously prescribed home exercise program, four due to pregnancy or lactation, three due to severe cardiopulmonary disease, one due to active malignancy, and one due to uncontrolled psychiatric illness. The final sample comprised 71 patients (Fig. 1).

### Sociodemographic and clinical data

Sociodemographic characteristics included age, sex, educational level, employment status, and marital status. Clinical data included disease duration and current medication use.

### Exercise adherence

Exercise adherence was assessed using the Exercise Adherence Rating Scale (EARS), for which Turkish validity and reliability have been established [14, 15]. The EARS was developed to evaluate adherence to prescribed exercise programs and consists of three sections (A, B, and C).

- **Section A** includes semi-structured questions with predefined categorical response options assessing exercise type, prescribed and actual exercise frequency and duration, and reasons for non-adherence.
- **Section B** consists of six items evaluating exercise-related behaviors, rated on a 5-point Likert scale from 0 (“strongly disagree”) to 4 (“strongly agree”). The total score ranges from 0 to 24, with higher scores indicating better exercise adherence.
- **Section C** comprises 10 items assessing facilitators and barriers related to exercise, also rated on a 5-point Likert scale (0–4). The total score ranges from 0 to 40, with higher scores indicating more positive attitudes and greater motivation.

The total EARS score was calculated by summing Sections B and C (B + C), with higher scores reflecting better overall exercise adherence.

### Perceived benefits and barriers

Perceived benefits and barriers to exercise were assessed using the Exercise Benefits/Barriers Scale (EBBS). The EBBS consists of 43 items, including 29 items in the benefits subscale and 14 items in the barriers subscale. The scale is rated on a 4-point Likert scale from 1 (“strongly agree”) to 4 (“strongly disagree”). The benefits subscale score ranges from 29 to 116, and the barriers subscale score ranges from 14 to 56. A higher benefits score indicates a more favorable perception of exercise. For the barriers subscale, lower scores indicate greater perceived barriers, whereas higher scores reflect fewer perceived barriers [16]. [

### Disease activity

Disease activity was evaluated using the Bath Ankylosing Spondylitis Disease Activity Index (BASDAI) [17]. The BASDAI consists of six items assessing fatigue, spinal pain, peripheral joint pain/swelling, enthesitis, and morning stiffness. Each item is rated on a visual analog scale (VAS) ranging from 0 (“none”) to 10 (“very severe”). The total score ranges from 0 to 10, with higher scores indicating greater disease activity.

### Functional status

Functional status was assessed using the Bath Ankylosing Spondylitis Functional Index (BASFI) [18]. The BASFI includes 10 items evaluating activities of daily living. Each item is rated on a VAS from 0 (“no difficulty”) to 10 (“very difficult”). The total score ranges from 0 to 10, with higher scores indicating worse functional status.

### Spinal mobility

Spinal mobility was assessed using the Bath Ankylosing Spondylitis Metrology Index (BASMI-2) [19]. The BASMI-2 includes five clinical measurements: tragus-to-wall distance, modified Schober test, lumbar lateral flexion, intermalleolar distance, and cervical rotation. Each parameter is scored from 0 to 2, yielding a total score ranging from 0 to 10. Higher scores indicate greater spinal mobility limitation.

### Pain

Pain intensity was measured using a Visual Analog Scale (VAS) assessing overall pain during the previous week. The scale ranges from 0 (“no pain”) to 10 (“unbearable pain”).

### Data collection procedure

Data were collected in two stages. In the first stage, sociodemographic and clinical characteristics were recorded, a standard physical examination was performed, and BASMI-2 measurements were obtained. In the same session, disease activity, functional status, and pain intensity were assessed using the BASDAI, BASFI, and VAS, respectively.

In the second stage, participants completed the EARS and EBBS questionnaires using a self-report method.

### Statistical analysis

Statistical analyses were performed using IBM SPSS Statistics version 26.0 (IBM Corp., Armonk, NY, USA). Continuous variables were presented as mean ± standard deviation or median (minimum–maximum), as appropriate, and categorical variables as frequencies and percentages. Normality of distribution was assessed using the Shapiro–Wilk test. For non-normally distributed variables, Spearman’s rank correlation coefficient was used in correlation analyses.

Multiple linear regression analysis was performed to identify variables independently associated with exercise adherence (total EARS score, B + C). Sociodemographic and clinical variables considered clinically relevant were included in the initial model, and backward elimination was applied for model selection. Variables with *p* > 0.05 were removed from the model.

Assumptions of linearity, normality of residuals, and homoscedasticity were examined to assess model validity. Multicollinearity was evaluated using the Variance Inflation Factor (VIF), with VIF < 5 considered acceptable. A p value < 0.05 was considered statistically significant in all analyses.

## Results

A total of 71 patients were included in this study, with a mean age of 38.73 ± 12.43 years. The distribution of sociodemographic characteristics is presented in Table 1. The majority of participants were university graduates (49.3%), employed (80.3%), male (62.0%), and married (73.2%).

**Table 1:** Distribution of sociodemographic characteristics of the patient group

	Results (*n* = 71)
Age (years), mean ± SD	38.73 ± 12.43
*Education Level, n(%)*	
Primary school	17 (23.9%)
High school	19 (26.8%)
University	35 (49.3%)
*Employment status, n (%)*	
Employed	57 (80.3%)
Unemployed	14 (19.7%)
*Gender, n (%)*	
Male	44 (62.0%)
Female	27 (38.0%)
*Marital status, n (%)*	
Married	52 (73.2%)
Single	19 (26.8%)

Data are presented as mean ± standard deviation (SD) or number (percentage)

Disease-related characteristics and medication use are presented in Table 2. The mean disease duration was 8.65 ± 6.42 years, with a median of 6 (2–22) years. The median (min–max) scores for VAS, BASDAI, BASFI, and BASMI-2 were 4 (0–9), 3.9 (0.4–10), 2 (0–9.2), and 2 (0–10), respectively. The most commonly used medication was anti-TNF agents (47.9%), followed by NSAIDs (39.4%).

**Table 2:** Disease-related factors and medications used in the patient group

	Result (*n* = 71)	
Disease-related factors	Mean ± SD	Median (min–max)
Disease duration (years)	8.65 ± 6.42	6 (2–22)
VAS	3.92 ± 2.42	4 (0–9)
BASDAI	4.06 ± 2.28	3.9 (0.4–10)
BASFI	2.60 ± 2.25	2 (0–9.2)
BASMI-2 total	2.80 ± 2.27	2 (0–10)

Medications used	n (%)
Anti-TNF	34 (47.9%)
NSAID	28 (39.4%)
DMARD	7 (9.9%)
IL-17 inhibitor	2 (2.8%)

Data are presented as mean ± standard deviation (SD) or number (percentage). VAS = Visual Analog Scale; BASDAI = Bath Ankylosing Spondylitis Disease Activity Index; BASFI = Bath Ankylosing Spondylitis Functional Index; BASMI = Bath Ankylosing Spondylitis Metrology Index

The analysis of EARS Section A items is presented in Table 3. The most frequently prescribed exercise type was general regular exercise (47.9%), followed by individualized home-based exercise programs (36.6%). The majority of participants were advised to exercise daily (88.7%), and 90.1% were recommended to maintain a continuous exercise program. According to self-reports, 31.0% of patients indicated that they did not perform any exercise. The most commonly reported reasons for non-adherence were daily responsibilities (19.7%) and lack of motivation/unwillingness (19.7%), followed by fatigue (11.3%) and pain (4.2%).

**Table 3:** Exercise Adherence Rating Scale (EARS) Section A Responses and EARS and EBBS Scores in the Patient Group

	Result (*n* = 71)	
EARS-A		
*A1. Recommended Exercise Type, n (%)*		
Individual sessions with a healthcare professional	8 (11.3)	
Individualized home exercise program	26 (36.6)	
Regular exercise in general	34 (47.9)	
Walking	1 (1.4)	
Being active in daily life	2 (2.8)	
*A2. Recommended Frequency, n (%)*		
Every day	63 (88.7)	
4–6 days per week	3 (4.2)	
2–3 days per week	5 (7.0)	
*A3. Recommended Duration, n (%)*		
Continuous	64 (90.1)	
For a specific period	7 (9.9)	
*A4. Actual Exercise Frequency, n (%)*		
Every day	19 (26.8)	
4–6 days per week	5 (7.0)	
2–3 days per week	18 (25.4)	
Once per week	7 (9.9)	
Never	22 (31.0)	
*A5. Reasons for Exercise Non-Adherence, n (%)*		
Daily responsibilities	14 (19.7)	
Lack of motivation/laziness	14 (19.7)	
Fatigue	8 (11.3)	
Pain	3 (4.2)	
EARS-B, mean ± SD, median (min–max)	10.96 ± 5.79	10 (1–24)
EARS-C, mean ± SD, median (min–max)	22.52 ± 5.76	23 (12–39)
EARS total, mean ± SD, median (min–max)	33.48 ± 10.81	34 (13–59)
EBBS-barriers, mean ± SD, median (min–max)	37.35 ± 8.35	37 (18–56)
EBBS-benefits, mean ± SD, median (min–max)	88.41 ± 13.70	84 (71–116)

Data are presented as number (percentage) or mean ± standard deviation (SD) and Median (Min-Max). EARS= Exercise Adherence Rating Scale; EBBS = Exercise Benefits and Barriers Scale

The distribution of EARS and EBBS scores is also shown in Table 3. The median (min–max) scores for EARS-B, EARS-C, and total EARS were 10 (1–24), 23 (12–39), and 34 (13–59), respectively. The median (min–max) scores for EBBS–Barriers and EBBS–Benefits were 37 (18–56) and 84 (71–116), respectively.

Spearman correlation analysis examining the relationship between exercise adherence and clinical parameters is presented in Table 4. Moderate positive and statistically significant correlations were observed between EARS-B, EARS-C, and total EARS scores and EBBS–Benefits scores (*r* = 0.636, *r* = 0.504, and *r* = 0.608, respectively; *p* < 0.001). No statistically significant associations were found between EARS scores and BASMI-2, BASDAI, BASFI, or VAS scores (*p* > 0.05).

**Table 4:** Spearman correlation analysis between exercise adherence (EARS), clinical parameters, and exercise benefit/barrier perceptions

Result (*n* = 71)			
Variable 1	Variable 2	Spearman *R*	*P* value
EARS B	BASMI-2 total	− 0.181	0.132
EARS B	EBBS-benefits	0.636*	**<0.001**
EARS B	EBBS-barriers	0.213	0.074
EARS B	BASDAI	0.020	0.871
EARS B	BASFI	0.130	0.281
EARS B	VAS	− 0.119	0.322
EARS C	BASMI-2 total	− 0.113	0.347
EARS C	EBBS-benefits	0.504*	**<0.001**
EARS C	EBBS-barriers	0.183	0.127
EARS C	BASDAI	0.015	0.902
EARS C	BASFI	0.083	0.494
EARS C	VAS	− 0.083	0.491
EARS Total	BASMI-2 total	− 0.180	0.134
EARS Total	EBBS-benefits	0.608*	**<0.001**
EARS Total	EBBS-barriers	0.198	0.098
EARS Total	BASDAI	− 0.026	0.828
EARS Total	BASFI	0.065	0.592
EARS Total	VAS	− 0.133	0.268

Spearman rank-order correlation analysis was used. Bold values indicate statistically significant results. *p < 0.05

EARS = Exercise Adherence Rating Scale; EBBS = Exercise Benefits and Barriers Scale; BASDAI = Bath Ankylosing Spondylitis Disease Activity Index; BASFI = Bath Ankylosing Spondylitis Functional Index; BASMI = Bath Ankylosing Spondylitis Metrology Index; VAS = Visual Analog Scale

Multiple linear regression analysis was performed to identify predictors of the total EARS score (Table 5). BASMI-2 total score (β = −1.73, *p* = 0.001), EBBS–Benefits score (β = 0.463, *p* < 0.001), and disease duration (β = 0.423, *p* = 0.019) were independently associated with exercise adherence. The model was statistically significant and explained 37.6% of the variance in exercise adherence (adjusted R² = 0.376; F(3, 67) = 15.04; *p* < 0.001).

**Table 5:** Multiple linear regression analysis predicting exercise adherence (EARS total score) in the patient group

Result (*n* = 71)					
Predictor variable	β	Standard error	95% CI	*p*	VIF
Intercept	− 6.23	6.79	(− 19.79, 7.32)	0.362	–
BASMI-2 total	− 1.73*	0.50	(− 2.74, − 0.73)	**0.001**	1.26
EBBS-benefits	0.463*	0.08	(0.31, 0.61)	**<0.001**	1.03
Disease duration	0.423*	0.18	(0.07, 0.78)	**0.019**	1.23

β coefficients are unstandardized regression coefficients.

Bold values indicate statistically significant results.

*p < 0.05. VIF = Variance Inflation Factor; EARS = Exercise Adherence Rating Scale; EBBS = Exercise Benefits and Barriers Scale; BASMI = Bath Ankylosing Spondylitis Metrology Index. Adjusted R² = 0.376; F(3, 67) = 15.04; p < 0.001.

## Discussion

In this cross-sectional study, factors associated with exercise adherence in patients with AS were examined. A significant association was observed between exercise adherence and perceived benefits of exercise. An inverse relationship was found between reduced spinal mobility and exercise adherence. Additionally, a positive association was identified between disease duration and exercise adherence. In contrast, no significant relationships were observed between exercise adherence and disease activity, functional status, or pain intensity.

AxSpA predominantly affects young adult males, and most patients are of working age [20]. Consistent with the literature, the mean age of our cohort was within the young adult range, and the majority of participants were male (62.0%) and employed (80.3%). Despite a relatively high educational level, exercise adherence remained at a moderate level, and 31% of patients reported not engaging in any exercise. This finding suggests that knowledge alone may not be sufficient to translate into sustained exercise behavior. Our results are consistent with studies indicating that physical activity levels in axSpA remain below recommended guideline targets. Carbo et al. reported that only 34–37% of patients with axSpA in two large Dutch cohorts met the World Health Organization recommendations for both aerobic and muscle-strengthening components of physical activity [21]. Similarly, Ma et al. found that 78.9% of outpatients with AS demonstrated low adherence to exercise therapy [22]. In our study, the proportion of patients reporting no exercise (31%) and the moderate total EARS score further indicate that exercise adherence in AS remains suboptimal despite routine clinical recommendations. Notably, although the vast majority of participants were advised to exercise daily and continuously, a marked discrepancy was observed between prescribed recommendations and actual practice, highlighting a gap between clinical advice and real-world behavior.

A positive association was identified between exercise adherence and perceived benefits of exercise, consistent with previous findings. Fongen et al. reported that 96% of patients believed physical activity had beneficial effects on health, with 37% perceiving improved disease stability and 33% reporting pain reduction [23]. Likewise, Lee et al. demonstrated that one of the most important facilitators of physical activity was awareness of its health benefits (68.9%) [24]. In our study, perceived exercise benefits were independently associated with exercise adherence, suggesting that cognitive factors may play a critical role in the initiation and maintenance of exercise behavior.

Randomized controlled trials and systematic reviews have demonstrated that patient education and behavior change–based interventions can improve exercise adherence in chronic rheumatic diseases [25–27]. In this context, Song et al. conducted a theory-based mHealth intervention grounded in the Health Belief Model, delivering individualized education sessions and regular informational content via the WeChat platform. The intervention resulted in improvements in disease knowledge, self-efficacy, and exercise adherence [28]. The authors emphasized that the increase in adherence was particularly associated with improvements in knowledge and self-efficacy. Although the cross-sectional design of our study precludes causal inference, the observed association between perceived exercise benefits and adherence supports the notion that cognitive perceptions constitute an important component of exercise behavior.

In axial spondyloarthritis, exercise is strongly supported not only for reducing disease activity but also for improving spinal mobility, functional status, and quality of life, and regular exercise is emphasized as a core component of disease management in the ASAS–EULAR recommendations [2]. Wang et al., evaluating a wearable-supported home-based exercise program, reported that regular and monitored exercise was associated with improvements in BASDAI, BASFI, and BASMI scores and that high adherence rates paralleled favorable clinical outcomes [29]. Similarly, Zimba et al. demonstrated that although most patients believed physical activity positively influenced disease progression, regular exercise rates remained low [10]. Additionally, Sarac et al. reported associations between perceived exercise benefits, kinesiophobia, and disease-related clinical parameters [15]. In our study, the moderate level of exercise adherence and the association with cognitive perception variables align with literature suggesting that positive beliefs about exercise do not consistently translate into sustained behavioral engagement. These findings indicate that prescribing exercise alone may be insufficient and that individual perceptions, motivation, and behavior change processes may play a substantial role in adherence.

Preservation of spinal mobility in axSpA is critically important for maintaining physical function and quality of life, and progressive mobility loss has been shown to negatively affect functional capacity and daily activities [30]. Chronic inflammation and accompanying structural changes may lead to reduced spinal flexibility and restricted range of motion, thereby limiting physical performance and activity levels [31]. In a systematic review examining determinants of exercise adherence, McDonald et al. reported that physical capacity and functional status may be associated with exercise behavior and that structural or physical limitations could influence adherence [32]. Although no significant association between BASMI and exercise adherence was detected in the correlation analysis of our study, BASMI-2 total score emerged as an independent predictor of exercise adherence in the multiple linear regression model. This finding suggests that objectively measured spinal mobility may be associated with exercise behavior. While the cross-sectional design does not allow causal interpretation, spinal mobility may represent a clinically relevant parameter to consider when evaluating exercise adherence. Individualized and graded exercise programs tailored to mobility levels may therefore be clinically meaningful.

In chronic inflammatory arthritis, supporting patients’ coping strategies and self-management skills is considered a core component of patient-centered care [33]. In individuals with longer disease duration, greater disease experience and symptom awareness may promote engagement in lifestyle modifications. Sang et al. reported that exercise adherence may be associated not only with sociodemographic factors but also with disease duration [34]. Similarly, our findings demonstrated a significant positive association between disease duration and exercise adherence, suggesting that longer disease duration may be one of the clinical variables related to adherence.

This study has several limitations. First, due to its cross-sectional design, the directionality and temporal sequence of associations cannot be established. Exercise adherence and physical activity levels were assessed using self-report instruments, introducing potential recall and social desirability bias. The single-center design and relatively limited sample size may restrict generalizability. Furthermore, psychological factors such as depression, self-efficacy, and kinesiophobia were not comprehensively assessed and may represent unmeasured variables influencing exercise adherence.

## Conclusion

In this cross-sectional study, exercise adherence among patients with AS was found to be moderate, and a substantial proportion of patients reported not engaging in exercise. Exercise adherence was particularly associated with perceived exercise benefits and appeared to be related to clinical variables such as spinal mobility and disease duration. These findings support the importance of moving beyond simple exercise prescription in clinical practice and emphasize the need for individualized, sustainable exercise approaches targeting cognitive perceptions and motivational processes, tailored according to mobility status. Future studies with larger samples, incorporating objective physical activity measurements and longitudinal or interventional designs including behavior change components, are warranted to further elucidate the determinants of exercise adherence.

## Supplementary Information

Below is the link to the electronic supplementary material.

Supplementary file1
