# Supplementary material for: Exercise adherence, perceived exercise benefits and barriers, and spinal mobility in ankylosing spondylitis: a cross-sectional study
Source: Rheumatol Int. 2026 Apr 24;46(5):80. doi: 10.1007/s00296-026-06118-z (PMC13106261; doi:10.1007/s00296-026-06118-z)
Supplement: Supplementary file 1 — Supplementary file1 [file 296_2026_6118_MOESM1_ESM.doc]

STROBE Statement—Checklist of items that should be included in reports of ***cross-sectional studies***

|  | Item No | Recommendation |
| --- | --- | --- |
| **Title and abstract** | 1 | (*a*) Indicate the study’s design with a commonly used term in the title or the abstract Page No: 1 (Title and Abstract) |
|  |  | (*b*) Provide in the abstract an informative and balanced summary of what was done and what was found Page No: 1 (Abstract) |
| Introduction | | |
| Background/rationale | 2 | Explain the scientific background and rationale for the investigation being reported Page No: 1–2 (Introduction) |
| Objectives | 3 | State specific objectives, including any prespecified hypotheses Page No: 2 (Introduction, last paragraph) |
| Methods | | |
| Study design | 4 | Present key elements of study design early in the paper Page No: 3 (Study Design) |
| Setting | 5 | Describe the setting, locations, and relevant dates, including periods of recruitment, exposure, follow-up, and data collection Page No: 3 (Study Setting and Participants) |
| Participants | 6 | (*a*) Give the eligibility criteria, and the sources and methods of selection of participants Page No: 3 (Study Setting and Participants) |
| Variables | 7 | Clearly define all outcomes, exposures, predictors, potential confounders, and effect modifiers. Give diagnostic criteria, if applicable Page No: 4–5 (Variables and measurement sections) |
| Data sources/ measurement | 8* | For each variable of interest, give sources of data and details of methods of assessment (measurement). Describe comparability of assessment methods if there is more than one group Page No: 5–6 (EARS, EBBS, BASDAI, BASFI, BASMI, VAS) |
| Bias | 9 | Describe any efforts to address potential sources of bias Page No: 8 (Discussion, Limitations) |
| Study size | 10 | Explain how the study size was arrived at Page No: 3 (Sample Size Calculation) |
| Quantitative variables | 11 | Explain how quantitative variables were handled in the analyses. If applicable, describe which groupings were chosen and why Page No: 5 (Statistical Analysis) |
| Statistical methods | 12 | (*a*) Describe all statistical methods, including those used to control for confounding Page No: 5 (Statistical Analysis) |
|  |  | (*b*) Describe any methods used to examine subgroups and interactions Not applicable |
|  |  | (*c*) Explain how missing data were addressed Not reported |
|  |  | (*d*) If applicable, describe analytical methods taking account of sampling strategy Page No: 3 (Study Setting and Participants) |
|  |  | (*e*) Describe any sensitivity analyses Not applicable |
| Results | | |
| Participants | 13* | (a) Report numbers of individuals at each stage of study—eg numbers potentially eligible, examined for eligibility, confirmed eligible, included in the study, completing follow-up, and analysed Page No: 3 (Sample Size Calculation; Figure 1) |
|  |  | (b) Give reasons for non-participation at each stage Page No: 5 (Sample Size Calculation) |
|  |  | (c) Consider use of a flow diagram Page No: 17 (Figure 1) |
| Descriptive data | 14* | (a) Give characteristics of study participants (eg demographic, clinical, social) and information on exposures and potential confounders Page No: 6 (Results; Table 1–2) |
|  |  | (b) Indicate number of participants with missing data for each variable of interest Not reported |
| Outcome data | 15* | Report numbers of outcome events or summary measures Page No: 12-16 (Results; Tables 1–5) |
| Main results | 16 | (*a*) Give unadjusted estimates and, if applicable, confounder-adjusted estimates and their precision (eg, 95% confidence interval). Make clear which confounders were adjusted for and why they were included Page No: 16 (Results; Table 5) |
|  |  | (*b*) Report category boundaries when continuous variables were categorized Not applicable |
|  |  | (*c*) If relevant, consider translating estimates of relative risk into absolute risk for a meaningful time period Not applicable |
| Other analyses | 17 | Report other analyses done—eg analyses of subgroups and interactions, and sensitivity analyses Page No: 6 (Correlation and regression analyses) |
| Discussion | | |
| Key results | 18 | Summarise key results with reference to study objectives Page No: 6 (Discussion, first paragraph) |
| Limitations | 19 | Discuss limitations of the study, taking into account sources of potential bias or imprecision. Discuss both direction and magnitude of any potential bias Page No: 8 (Discussion, Limitations) |
| Interpretation | 20 | Give a cautious overall interpretation of results considering objectives, limitations, multiplicity of analyses, results from similar studies, and other relevant evidence Page No: 7–8 (Discussion) |
| Generalisability | 21 | Discuss the generalisability (external validity) of the study results Page No: 8 (Discussion, Limitations) |
| Other information | | |
| Funding | 22 | Give the source of funding and the role of the funders for the present study and, if applicable, for the original study on which the present article is based Title Page |

*Give information separately for exposed and unexposed groups.

**Note:** An Explanation and Elaboration article discusses each checklist item and gives methodological background and published examples of transparent reporting. The STROBE checklist is best used in conjunction with this article (freely available on the Web sites of PLoS Medicine at http://www.plosmedicine.org/, Annals of Internal Medicine at http://www.annals.org/, and Epidemiology at http://www.epidem.com/). Information on the STROBE Initiative is available at www.strobe-statement.org.
